# Integrated vs non-integrated treatment outcomes in dual diagnosis disorders: A systematic review

**DOI:** 10.4102/hsag.v28i0.2094

**Published:** 2023-04-25

**Authors:** Ashley Chetty, Tharina Guse, Mosa Malema

**Affiliations:** 1 Department of Psychology, Faculty of Humanities, University of Pretoria, Pretoria, South Africa

**Keywords:** dual diagnosis, mental disorders, substance use disorder, alcohol dependence, integrated treatment, non-integrated treatment, service delivery, randomised controlled trials, systematic review

## Abstract

**Background:**

The incidence of dual diagnosis (DD) (i.e. substance use disorders [SUD] and co-occurring mental disorders) is widespread; however, they vary widely in permutation and combination. As a result, establishing effective and empirically supported interventions for this clinical population remains challenging.

**Aim:**

This study aimed to examine current literature on the treatment outcomes for patients with DD.

**Method:**

A systematic review of randomised controlled trials (RCTs) published between 2009 and 2018 was conducted for two broad intervention categories identified by the literature: non-integrated and integrated treatment. Multiple electronic databases were searched using the Preferred Reporting Items for Systematic Reviews and Meta-Analyses guidelines (PRISMA).

**Results:**

The search generated a total of 743 studies, of which 11 satisfied the inclusion criteria. These studies were thematically synthesised into two main analytical themes: ‘treatment outcomes’ and ‘reported strengths and limitations of DD treatment’. Specifically, integrated treatment held an advantage over non-integrated treatment in significantly improving psychiatric symptomatology. However, no significant benefits were found between integrated and non-integrated treatment regarding substance misuse and treatment retention.

**Conclusion:**

Overall, the results provided insufficient evidence to support the enhanced efficacy of integrated or non-integrated treatment over the other in treating patients with DD.

**Contribution:**

The study’s findings were used to provide recommendations to inform the clinical psychological service delivery of dual diagnosis treatment in South Africa and also to identify gaps in the literature and highlight areas for future research.

## Introduction

Over the past decade, the incidence of a diagnosed mental disorder along with a co-occurring substance use disorder (SUD) in the same person, referred to as dual diagnosis (DD), has become a well-established and evolving field of research (Iudici et al. 2020; Morisano, Babor & Robaina 2014; Vitali et al. 2018). Recent findings found that up to 75% of patients with a severe mental illness have also been diagnosed with an SUD, while 60% of adult patients with an SUD were found to be diagnosed with at least one severe mental illness (Temmingh et al. 2018). The prevalence of DD is being increasingly studied in the South African context (Pasche et al. 2015). For example, the South African Community Epidemiology Network of Drug Use (SACENDU 2021) found that as of December 2019, 15% of the total sample of participants presented with DD at treatment admission. Additionally, the South African Stress and Health study, conducted between 2002 and 2004, determined that 21.3% of those with a lifetime SUD also suffered from a psychiatric disorder (Saban et al. 2014).

Currently, there are no diagnostic criteria for DD or co-occurring disorders included in the *Diagnostic and Statistical Manual for Mental Disorders*, fourth edition (DSM-IV) or fourth edition-text revision (DSM-IV-TR). The DSM-V utilises a categorical approach but proposes a “dimensional” approach that allows for a more flexible understanding of DD that attempts to accommodate the subjectivity of each patient and has the potential to improve the clinical practice and diagnostic accuracy (Vitali et al. 2018). However, due to the high prevalence of this psychiatric presentation, standardised diagnostic criteria need to be developed and added to newer editions of the DSM to assist clinicians in the effective and timely diagnosis and treatment of DD patients (Iudici et al. 2020).

Patients with DD present with significantly complex clinical profiles (Iudici et al. 2020). When compared to patients with single morbidities, patients with DD present with higher rates of treatment non-compliance and relapse (Horsfall et al. 2009), lower levels of motivation to change, reduced treatment engagement, poor adaptive coping skills (Priester et al. 2016), increased psychiatric morbidities, impaired quality of life (McCallum et al. 2015; Morojele, Saban & Seedat 2012), and severely compromised socio-economic functioning (Vitali et al. 2018). As a result, DD has been associated with a poorer long-term prognosis (Kay-Lambkin, Baker & Lewin 2004). Thus, it becomes increasingly difficult for treatment programmes to sufficiently address the diverse challenges of this clinical population. Moreover, to implement effective treatment strategies, service providers require a firm understanding of DD and appreciation for the current evidence (Adams et al. 2016).

One of the overarching contestations remains the nature of the interaction between mental disorders and SUDs. Literature suggests that no single model can account for the heterogeneity of patients who present with DD (Iudici et al. 2020). Thus, several frameworks have emerged that provide differing insights into the potential connections. For example, the common factors model suggests that DD is understood as an expression of underlying genetic vulnerabilities, such as a predisposition, cognitive functioning or liability for substance dependency, which leaves individuals more susceptible to developing SUDs (Morisano et al. 2014; Mueser, Drake & Wallach 1998). Similarly, the secondary SUD models assume that being diagnosed with a severe mental disorder increases a patient’s vulnerability to developing a co-occurring SUD. Specifically, it is posited that patients with severe mental disorders use substances to alleviate pain and bouts of intense dysphoria (Mueser et al. 1998). The secondary psychiatric illness model considers that a patient with an SUD becomes vulnerable to developing a severe mental disorder as minor symptoms of mental illness become exacerbated until diagnosable (Morisano et al. 2014). These presentations are usually understood to be substance-induced mental health disorders. Lastly, the bidirectional model suggests that DD is maintained by a consistent and ongoing interaction between the severe mental disorder and SUD (Morisano et al. 2014; Mueser et al. 1998). Therefore, one disorder serves to worsen or maintain the other and vice versa.

Consequently, a continuum of care has evolved to accommodate the varying conceptualisations of DD. The literature has identified two broad intervention categories: non-integrated and integrated treatment. Non-integrated treatment generally describes the separate treatment of co-occurring conditions in the context of patients with DD (Morisano et al. 2014). This approach maintains a clear delineation of professional boundaries and relies on little to no co-ordination between service providers (Brousselle et al. 2012). Non-integrated treatment can be further differentiated into two approaches: sequential and parallel treatment.

Firstly, sequential treatment manages patients by systematically addressing one condition at a time, usually to efficiently focus efforts and resources towards long-term recovery and rehabilitation (Horsfall et al. 2009; Sterling, Chi & Hinman 2011). Some argue that it is imperative first to address their mental disorder, prioritise the development of adaptive coping strategies, and then address their substance misuse (Morisano et al. 2014). Others suggest it is better first to address the SUD to manage the substance use and assure greater psychotherapeutic and pharmacological compliance moving forward (McCauley et al. 2012). For instance, Green et al. (2015) identified that for participants: (1) learning about the effects of substances increased their motivation to remain abstinent, (2) achieving abstinence further motivated them to meaningfully address their mental health concerns, and (3) maintaining their abstinence increased their self-confidence, sense of agency, and level of functioning.

Secondly, parallel treatment allows for the treatment of both the SUD and mental disorder by utilising different service providers for each disorder who work in an uncoordinated fashion (Horsfall et al. 2009; Morisano et al. 2014). The existing literature suggests that there are mixed results regarding the effectiveness of this model. Mangrum, Spence and Lopez (2006) found that integrated treatment led to greater reductions in psychiatric hospitalisation and arrest frequency compared to a parallel treatment condition. Similarly, Randall, Thomas and Thevos (2001) compared an integrated treatment group and non-integrated control group with a sample of patients diagnosed with a social anxiety disorder and an alcohol use disorder. The results indicated that both groups experienced improvements in their alcohol misuse behaviours and social anxiety symptoms. Notably, at post-treatment, the treatment group was drinking more frequently and reported heavier drinking days than the control group.

In contrast, integrated treatment describes the simultaneous treatment of an individual’s SUD and psychiatric disorder that maintains coordinated interaction between service providers (Horsfall et al. 2009; Morisano et al. 2014; Sinha, Garg & Prakash 2018). Treatment is usually carried out by the same clinician or a multidisciplinary team of clinicians where knowledge and expertise are shared to enhance the effectiveness of treatment. In general, integrated treatment is considered the preferred model as its outcomes generally outperform those of non-integrated treatment (Back et al. 2019; Priester et al. 2016; Sinha et al. 2018). However, there remains reservation regarding the feasibility of implementing integrated treatment (Cleary et al. 2009). For example, Cleary et al. (2009) conducted a comprehensive review of 25 randomised controlled trials (RCTs) comparing psychosocial interventions for substance misuse in patients with a severe mental illness. No significant advantages were found between the groups with regard to substance misuse. The study suggested that effective treatment relied on addressing a patient’s sense of personal control, self-confidence, place of belonging, commitment to change, and hope for their future. The researchers did identify high drop-out rates that needed to be considered when interpreting the results.

Despite the rapid development in DD-centric treatment programmes over the past decade, there remain few validated treatment options and limited evidence to support the efficacy of specific psychological interventions (Fantuzzi & Mezzina 2020; Kay-Lambkin et al. 2004; Morojele et al. 2012; Pasche et al. 2015). Specifically, there are a lack of published studies that extensively explore the treatment-related outcomes of DD-centric care in South Africa. Therefore, it remains unclear to what extent the existing literature has informed current practices for treating DD in South Africa. These gaps in the literature illustrate the need for specific research that operationalises the recent findings on DD treatment for the South African context.

Consequently, this study aimed to conduct a systematic review of RCTs for the integrated and non-integrated treatment outcomes for patients with DD. The study’s objectives were to: (1) summarise the treatment outcomes of integrated and non-integrated interventions, (2) summarise the strengths and limitations of integrated and non-integrated treatment, and (3) propose evidence-based recommendations to inform the clinical psychological service delivery of DD-focused treatment in South Africa.

## Methodology

### Research design

This study followed a descriptive research question determined to explore the status of knowledge regarding the integrated and non-integrated treatment outcomes for patients with DD disorders. Informed by the PRISMA, a systematic review was conducted to identify, critically appraise, and summarise a selection of high-quality relevant studies (Page et al. 2021b).

### Inclusion and exclusion criteria

The following criteria were used to select studies for review:

- Only studies that used a RCT design to evaluate the treatment outcomes of integrated or non-integrated interventions for patients with DD were included. Other research designs such as controlled studies that did not use randomisation, pre–post evaluations with no clear control condition present, case studies and secondary analyses were excluded.
- Only studies published between 2009 and 2018 to ensure that contemporary evidence, at the time of writing, was reviewed, and the results of this review were temporally relevant.
- Only studies with adult samples of participants aged 18 years or older were included. This criterion was motivated by findings from the SACENDU (2021), which noted significant age differences for specific substance users across their treatment sites. For example, persons whose primary substance of use was alcohol, crack/cocaine, cannabis/mandrax or over-the-counter prescription medications were substantially older (> 30 years old). By contrast, persons whose primary substances of use were inhalants and cannabis tended to be younger (< 30 years old). Therefore, to not limit the breadth of this review and accommodate for the heterogeneous nature of this clinical population, a broader age restriction was implemented.
- Only studies with participants who met the standard diagnostic criteria for DD (i.e. the presence of a SUD and co-occurring mental disorder in the same person) as verified by the American Psychiatric Association’s *Diagnostic and Statistical Manual of Mental Disorders*, fourth edition (DSM-IV) were included.

### Search strategy

An exhaustive literature search of the following electronic databases was performed: Scopus, ScienceDirect, Google Scholar, and EbscoHost (including Academic Search Complete, Africa-Wide Information, APA PsycARTICLES, APA PsycINFO, CINAHL, Family & Society Studies Worldwide, Health Source: Nursing/Academic Edition, Humanities Source, MasterFILE Premium, and MEDLINE). Each research platform was electronically searched using the following search terms: ‘dual diagnosis’, ‘co-occurring disorders’, ‘integrated treatment/intervention’, ‘non-integrated treatment/intervention’, ‘sequential treatment/intervention’, ‘parallel treatment/intervention’, ‘randomised control trial’, and ‘adult’. In addition, Boolean operators and specific limiters were utilised to refine the search procedure.

An initial search of the databases yielded 708 potentially relevant studies. The first author perused the reference lists of the various articles to collect more relevant studies that may not have surfaced during the initial search. This resulted in a total of 743 studies, of which 15 duplicates were removed and the remaining studies were then subjected to a primary screening phase.

### Study selection and quality assessment

In the primary screening phase, the first author examined the various studies’ titles and abstracts to assess their relevance. Subsequently, 616 studies were excluded for being methodologically or contextually unrelated to the research question. Following this, 112 studies remained. During the secondary screening phase, a more in-depth reading was conducted by two reviewers (the first and second authors), while a third reviewer (the third author) was available to consult should there be a disagreement between the first two reviewers. To reduce bias in the study selection process and to ensure that the appraisal was rigorous, studies were appraised by the two reviewers independently using the same critical appraisal tools.

**FIGURE 1:**
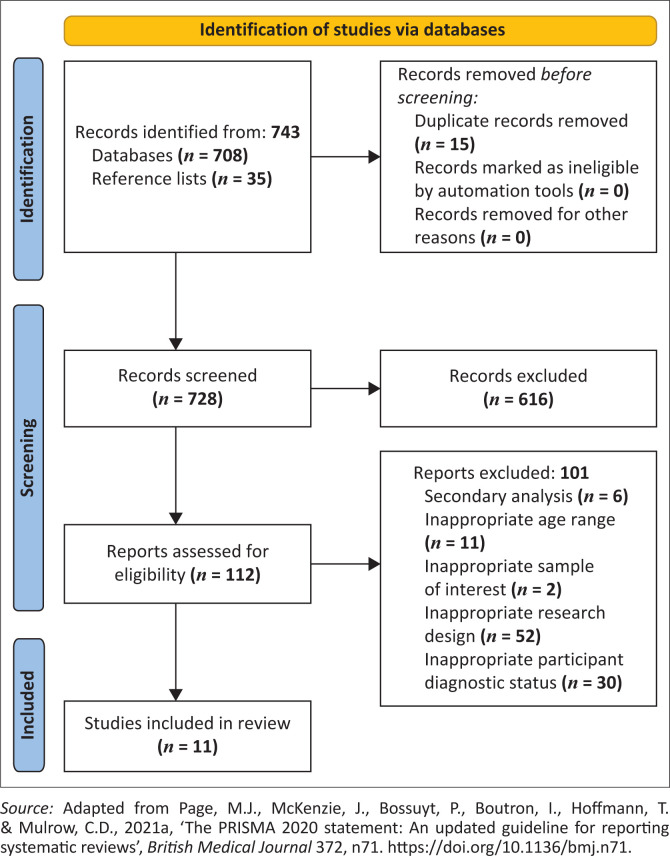
Flow diagram of the search procedure.

The studies were then subject to a quality assessment phase. This phase was concerned with determining whether the prospective studies’ research designs were: (1) valid and methodologically sound to be considered an RCT and (2) whether the reported outcomes were reliable and locally applicable. Specifically, the Critical Appraisal Skills Programme (CASP 2020) checklist for RCTs was used to assess the quality of the eligible studies. Figure 1 presents a flow diagram detailing the study selection procedure.

### Data extraction and analysis

The data extraction process required that the authors first become familiar with the full-text articles of each study. Following multiple readings, the detailed characteristics of the 11 studies were extracted and are documented in Table 1. Each study was systematically analysed in terms of their potential relevance and applicability to the main concepts of this systematic review. An abundance of descriptive data was extracted, which formed the basis for the subsequent data analysis required to answer this research question.

**TABLE 1:** Characteristics of included studies.

Author/s	Objectives and outcomes measure	Study setting	Type of DD	Sample description	Study design and intervention	Main findings
Coffey et al. (2016) USA	Evaluate the efficacy of mPE, relative to the TSF, for treating PTSD with co-occurring substance dependence.	Community residential SUD treatment facility, in-patient.	PTSD + alcohol dependence	*n* = 126 $\bar{\text{x}}$ age = 34 years Gender: M – 53.97% F – 46.03% Race/ethnicity: W – 79.4% B – 19% O – 1.6%	**Experiment:** mPE + trauma-focused motivational enhancement + TAU, *integrated* **Experiment:** mPE + TAU, *integrated* **Control:** HLS + TAU, *non-integrated*	Both experiment conditions: - achieved significantly improved PTSD outcomes than the control condition. - did not differ on PTSD symptoms at the end of treatment, three-, or six-month follow-up. - did not differ with substance use outcomes. Clinically significant improvement in trauma symptoms were reported for all conditions at the end-of-treatment.
Courbasson, Nishikawa & Dixon (2012) USA	Examine the preliminary efficacy of adapted DBT, relative to TAU, for treating co-occurring ED and SUD.	Specialised substance use and mental health clinic, out-patient.	ED + SUD	*n* = 25 $\bar{\text{x}}$ age = 32.53 years Gender: F – 100% Race/ethnicity: W – 100%	**Experiment:** DBT, *integrated* **Control:** TAU, *integrated*	Experiment condition observed better retention rates relative to the control at post-treatment and follow-up. Experiment condition had a significant positive effect on behavioural and attitudinal features of disordered eating, substance use severity and use, negative mood regulation, and depressive symptoms.
Foa et al. (2013) USA	Compare the efficacy of an evidence-based treatment for alcohol dependence and an evidence-based treatment for PTSD, their combination, and supportive counselling.	Center for the Treatment and Study of Anxiety and the Philadelphia Veterans Affairs Hospital, out-patient.	PTSD + alcohol dependence	*n* = 165 $\bar{\text{x}}$ age = 42.73 years Gender: M – 65.5 % F – 34.5% Race/ethnicity: W – 30.4% B – 63.6%; Hispanic – 4.2% Native American – 0.6% O – 1.2%	**Experiment:** PTSD exposure therapy + naltrexone, *non-integrated* **Experiment**: PTSD exposure therapy + pill placebo, *non-integrated* **Experiment:** Supportive counselling + naltrexone, *non-integrated* **Control:** Supportive counselling + pill placebo, *non-integrated*	All four conditions had significant reductions in the percentage of drinking days. However, those who received naltrexone had lower percentages of days drinking than those who received a placebo. All four conditions reported reductions in PTSD symptoms, but the main effect of prolonged exposure therapy was not statistically significant.
Garland et al. (2016) USA	Compare the effectiveness of MORE, relative to CBT and TAU, for treating previously homeless men residing in a therapeutic community.	Modified therapeutic community programme in an urban area, out-patient.	Psychiatric d/o + SUD	*n* = 180 $\bar{\text{x}}$ age = 37.63 years Gender: M – 100% Race/ethnicity: W – 42.2% B – 44.5% O – 13.3%	**Experiment:** MORE, *integrated* **Experiment:** CBT, *integrated* **Control:** TAU, *non-integrated*	MORE was associated with: - modest yet significant improvements in substance craving, post-traumatic stress, and negative affect than CBT. - more significant improvements in post-traumatic stress and positive affect than TAU. A significant indirect effect of MORE on decreasing craving and post-traumatic stress by increasing dispositional mindfulness was observed.
Gouzoulis-Mayfrank et al. (2015) Germany	Evaluate the efficacy of IT, relative to TAU, for treating DD patients.	Large psychiatric hospital, in-patient and out-patient.	Schizophrenia, schizophreniform, or schizoaffective d/o + substance misuse or dependence	*n* = 100 $\bar{\text{x}}$ age = 30.97 years Gender: M – 84% F – 16% Race/ethnicity: Not indicated	**Experiment:** IT, *integrated* **Control:** TAU, *non-integrated*	The experiment group developed higher motivation for abstinence than the control group and reduced their substance use to a greater extent than the control group. Global functioning and retention rate were also higher for the experiment group but did not reach significance.
Graham et al. (2016) UK	Assess the effectiveness and feasibility of BIMI, relative to TAU, for improving engagement in drug and alcohol misuse treatment.	Acute mental health hospital, in-patient.	Schizophrenia, schizoaffective or delusional d/o; bipolar affective d/o + alcohol and/or drug abuse/dependence	*n* = 59 $\bar{\text{x}}$ age = 38.6 years Gender: M – 84.75% F – 15.25% Race/ethnicity: W – 47.6% B – 25.35% Asian – 16.95% Mixed – 10.05%	**Experiment:** BIMI, *non-integrated* **Control:** TAU, *non-integrated*	85% of participants were retained in the study and 70% at follow-up meaningfully engaged in BIMI. Both conditions: - remained in the “low” readiness to change category for alcohol and drugs at follow-up. - reduced the number of days they used substances by more than half. However, the effect was not significant. No evidence of a treatment effect on HADS Anxiety. A modest but insignificant effect on the HADS Depression.
McGovern et al. (2011) USA	Determine the potential efficacy of ICBT, relative to IAC.	Community addiction treatment programmes, out-patient.	PTSD + SUD	*n* = 53 $\bar{\text{x}}$ age = 37.3 years Gender: M – 41.65% F – 58.35% Race/ethnicity: W – 91.35% O – 8.65%	**Experiment:** ICBT, *integrated* **Control:** IAC, *non-integrated*	Experiment condition was more effective than the control in reducing PTSD re-experiencing symptoms and PTSD diagnosis. Control condition was comparably effective to the experiment in substance use outcomes and psychiatric symptom severity. Participants in the control condition with severe PTSD were less likely to initiate and engage in the therapy than those in the experiment condition. In general, participants with severe PTSD were more likely to benefit from ICBT.
McGovern et al. (2015) USA	Assess the efficacy of ICBT, relative to IAC and standard care alone, on substance use and PTSD symptoms.	Addiction treatment agencies, out-patient.	PTSD + SUD	*n* = 221 $\bar{\text{x}}$ age = 35.3 years Gender: M – 40.7% F – 59.3% Race/ethnicity: W – 95.5% O – 4.5%	**Experiment:** ICBT + SC, *integrated* **Experiment:** IAC + SC, *non-integrated* **Control:** SC only, *non-integrated*	All conditions reported reductions in PTSD symptoms with no significant difference between them. ICBT produced: - better outcomes on toxicology than IAC or SC and had a more significant reduction in reported drug use than SC. - better therapy continuation versus IAC.
Mills et al. (2012) Australia	Evaluate the efficacy of COPE, relative to TAU, for PTSD and substance dependence symptom severity.	Substance use treatment services, out-patient.	PTSD + substance dependence	*n* = 103 $\bar{\text{x}}$ age =33.7 years Gender: M – 37.9% F – 62.1% Race/ethnicity: Australian-born– 84.5% Aboriginal – 5.8%	**Experiment:** COPE + TAU, integrated **Control:** TAU only, non-integrated	Both conditions reported significant reductions in PTSD symptom severity. However, the treatment group reported significantly greater reductions in PTSD symptom severity. No significant between-group difference found in: - improvement in the severity of substance dependence. - changes in substance use, depression, or anxiety.
Sannibale et al. (2013) Australia	Assess the efficacy of ICBT, relative to CBT for AUD only, for PTSD and co-existing AUD.	Mental health clinics, out-patient.	PTSD + AUD	*n* = 62 $\bar{\text{x}}$ age = 41.18 years. Gender: M – 47% F – 53% Race/ethnicity: Not indicated	**Experiment:** IT for PTSD + AUD, *integrated* **Control:** Alcohol support, *non-integrated*	Both conditions reported reductions in PTSD severity. Participants in experiment condition who received one or more exposure therapy sessions exhibited a twofold greater rate of clinically significant change in Clinician-Administered PTSD Scale severity at follow-up than in the control condition. Participants in the control condition reported more significant reductions than the experiment condition in alcohol consumption, dependence, and problems with treatment from other services at follow-up.
Wüsthoff, Waal & Gråwe (2014) Norway	Investigate the effectiveness of IT, relative to TAU, for SUD with co-occurring anxiety and/or depression.	Community mental health centres, out-patient.	Anxiety and/or depression + d/o of abuse or dependence from drugs and alcohol	*n* = 76 $\bar{\text{x}}$ age = 37.25 years Gender: M – 52.6% F – 47.4% Race/ethnicity: Norwegian – 95.8% O – 4.2%	**Experiment:** IT, *integrated* **Control:** TAU, *non-integrated*	Both conditions reduced their alcohol and substance use during the trial but observed no change in psychiatric symptoms in either group. The experiment condition reported a more significant increase in motivation for substance use treatment after 12 months than had the control condition.

DD, dual diagnosis; USA, United States of America; UK, United Kingdom; mPE, modified prolonged exposure; TSF, twelve-step facilitation therapy; PTSD, posttraumatic stress disorder; SUD, substance use disorder; M, male; F, female; W, White; B, Black; O, other; TAU, treatment as usual; HLS, health information-based control condition; DBT, dialectical behavioural therapy; ED, eating disorder; MORE, mindfulness-oriented recovery enhancement; CBT, cognitive behavioural therapy; d/o, disorder; IT, integrated treatment; BIMI, brief integrated motivational intervention; HADS, hospital anxiety and depression scale; ICBT, integrated cognitive behavioural therapy; IAC, individual addiction counselling; SC, standard care; COPE, concurrent treatment of PTSD and substance use disorders using prolonged exposure; AUD, alcohol use disorder.

Data analysis took place through the framework provided by thematic synthesis (Thomas & Harden 2008). The synthesis procedure was guided by three separate, although somewhat overlapping, stages. Stage one required the authors to become familiar with the descriptive data gathered in the data extraction phase. Stage two involved coding of the descriptive data line by line to generate ‘free codes’. The similarities and differences between these codes were identified and organised into descriptive themes that formed a hierarchical structure.

Following this, stage three was primarily concerned with ‘translating’ these descriptive themes into analytic themes. That is, engaging in an iterative process of taking descriptive themes from one study and recognising comparable concepts in another study (Thomas & Harden 2008). Therefore, pulling together the corroborating descriptive themes allowed for the development of an interpretation or ‘a line of argument’ that went beyond the content of the original studies and answers the research question. At this point, the researcher had to determine: (1) whether these analytic themes remained faithful to the data from which they were extracted and (2) whether any factors explain why an interpretation gained in one study cannot be transferred to another.

### Ethical considerations

This study obtained ethical approval from the Faculty of Humanities Research Ethics Committee at a large public university in Gauteng (Reference: HUM011/0519). Due to the nature of this systematic review, participant consent was not required as the authors consulted published and available literature in the public domain. Further, the authors observed the ethical standards required in terms of the University’s Code of Ethics for researchers and the Policy Guidelines for responsible research.

## Results

### Characteristics of studies

As indicated in Table 1, the study participants were predominantly male (54.5%), white (45.5%) and presented with a mean age ranging between 25 and 42.73 years. All 11 studies used standard diagnostic criteria, primarily the *DSM-IV*, to establish their participants’ diagnostic status. Post-traumatic stress disorder (PTSD) (54.5%) was the most prominent psychiatric diagnosis, followed by schizophrenia, schizophreniform or schizoaffective disorder (18.2%), and then one study each accounting for depressive and/or anxiety disorders, eating disorders, and an array of ‘psychiatric disorders’. Additionally, non-specific SUDs were the most prominent substance-related diagnoses, followed by alcohol dependence/use disorder (36.4%). A majority of studies (54.5%) were conducted in the United States of America. In contrast, two studies were conducted in Australia, one in the United Kingdom, one in Germany, and the remaining study in Norway.

Of the 11 studies that were retrieved, four studies conducted standard RCTs (36.4%), one performed a matched RCT, one a single-blind RCT, two pragmatic RCTs (18.2%), one stage I phase III RCT, and one three-group repeated-measure RCT. A majority of the studies (*n* = 9, 81.8%) were considered explanatory trials aimed at testing the efficacy of an intervention by determining whether it produces the expected result under ideal circumstances. The remaining two studies were pragmatic trials aimed at testing the effectiveness of an intervention by measuring the degree of beneficial effect in a more generalisable setting.

### Quality of included studies

All 11 studies used a treatment fidelity measure to ensure that their study and the chosen interventions were conducted in a reliable and trustworthy fashion. Each study utilised empirically validated clinician or self-reported measures (e.g. DSM, Structured Clinical Interview; Clinician-Administered PTSD Scale; Post-traumatic Stress Diagnostic Scale; Beck Depression Inventory and Alcohol Use Disorders Identification Test). Therefore, the remaining studies were of a moderate to high level of: (1) relevance to the context of the review’s research question and (2) scientific quality in relation to each study’s reported results and methodological procedures.

## Main findings

### Theme one: Treatment outcomes

Overall, the general trend of results found that integrated treatment outperformed non-integrated treatment in significantly improving the psychiatric symptomatology for participants with DD.

### Psychiatric symptomology

Specifically, a majority of studies (45.5%) found that integrated treatment produced significantly greater reductions in PTSD symptoms for patients with DD when compared to non-integrated treatment (Coffey et al. 2016; Garland et al. 2016; McGovern et al. 2011; Mills et al. 2012; Sannibale et al. 2013). Notably, irrespective of the treatment model utilised, the interventions that had the most significant impact on improving PTSD symptoms included dialectical behavioural therapy (DBT), mindfulness-oriented recovery enhancement (MORE), integrated cognitive behavioural therapy (ICBT), and concurrent treatment of PTSD and SUDs using prolonged exposure (COPE).

Two studies (18.2%) found that integrated treatment produced superior reductions in anxiety and depressive symptoms for participants with DD when compared to non-integrated treatment (Coffey et al. 2016; Garland et al. 2016). Three studies (27.3%) found no differences between integrated and non-integrated treatment in reducing anxiety and depressive symptoms for participants with DD (Graham et al. 2016; Mills et al. 2012; Wüsthoff et al. 2014).

Of the 11 studies included in this study, only one study reported the outcome of psychotic symptomatology (Gouzoulis-Mayfrank et al. 2015). Notably, the results indicated that both the global level of psychological functioning and psychotic symptoms improved for all participants in the study. However, for negative symptoms and general psychopathology, the study did not observe a significant between-group difference. Therefore, both integrated and non-integrated treatments produced similar reductions in the severity of psychotic symptoms in a sample of patients with a psychotic disorder and co-occurring substance dependence.

Similarly, of the 11 studies included in this study, only one study reported the outcome of ED-related behaviours (Courbasson et al. 2012). Preliminary support was afforded for integrated treatment’s superiority over non-integrated treatment in improving eating-related behaviours for participants with an ED and co-occurring SUD.

### Substance use symptomology

A majority of studies (54.5%) found that both integrated and non-integrated treatments evidenced similar reductions in substance use outcomes for participants with DD, with no significant between-group differences observed (Coffey et al. 2016; Gouzoulis-Mayfrank et al. 2015; Graham et al. 2016; McGovern et al. 2011; Mills et al. 2012; Wüsthoff et al. 2014). Similarly, interventions that had the most significant impact on substance use outcomes included DBT, COPE, and ICBT.

### Treatment retention, engagement and completion

Four studies determined that both integrated and non-integrated treatments reported similar retention rates with no significant between-group differences being observed (Coffey et al. 2016; Graham et al. 2016; McGovern et al. 2011). Two studies found that retention rates for integrated treatment were superior to non-integrated treatment (Courbasson et al. 2012; McGovern et al. 2015). Overall, these results suggest that both integrated and non-integrated treatments elicit similar retention rates among participants with DD, with no significant between-group differences observed. However, of the few studies reported on the measure, non-integrated treatment produced significantly higher completion rates than integrated treatment.

### Theme two: Reported strengths and limitations of dual diagnosis treatment

The findings suggest that therapeutic change was facilitated using cognitive behavioural therapy (CBT)-informed principles. For example, mindfulness strategies were incorporated into the programme to assist participants in developing self-regulatory skills (Courbasson et al. 2012). Exposure to dispositional mindfulness produced a considerable therapeutic effect as it reported enhanced participants’ mindful awareness, cognitive flexibility, and cognitive reappraisal (Garland et al. 2016). Moreover, maintaining strong lines of communication and co-ordination between the treatment providers was cited as powerful facilitators of change (Coffey et al. 2016). Similarly, participants found programmes that prioritised the therapeutic relationship and improved motivation for treatment significantly assisted their journey to recovery (Coffey et al. 2016; Wüsthoff et al. 2014).

Additionally, carrying out treatment in in-patient residential facilities appeared to assist in eliminating difficulties related to treatment attendance and retention, such as missed appointments due to family and childcare coverage, transportation challenges, and complicated work schedules (Coffey et al. 2016). Access to illicit substances was also limited and the risk of elevated substance cravings and subsequent relapse due to elevated stress and trauma-related negative affect were reduced (Coffey et al. 2016). Moreover, the ancillary support services embedded in the long-term therapeutic communities, such as housing and accommodation facilities, and vocational training offered participants additional advantage in their recovery journey (Garland et al. 2016; Mills et al. 2012). However, the nature of treatment in these in-patient residential facilities was cited as not easily translatable to out-patient care and remains a resource-heavy endeavour (Graham et al. 2016). For example, utilising specialist DD trained staff requires additional training and financial investment; however, the availability of these expert resources is limited and creates an additional level of organisational complexity (Coffey et al. 2016).

## Discussion

The broad research aim guiding this study was to systematically examine current literature on the integrated and non-integrated treatment outcomes for patients with DD. The primary aim of this study was to summarise treatment outcomes of integrated and non-integrated interventions for patients with DD. Firstly, integrated treatment evidenced significantly greater reductions in psychiatric symptomatology, particularly PTSD symptoms, compared to non-integrated treatment. This finding is consistent with previous research (Back et al. 2019; Mojtabai et al. 2014; Priester et al. 2016; Sinha et al. 2018).

Secondly, this review found that non-integrated and integrated treatment elicited comparable between-group improvement in substance use symptomatology. All the integrated programmes identified among the 11 studies included in this review addressed SUD simultaneously alongside psychiatric disorders, while a large proportion of the non-integrated treatments focused solely on substance abuse. Therefore, it is plausible that the two treatment models could observe equivalent substance use outcomes. There are significant findings declaring integrated treatment’s superiority to non-integrated treatment (Back et al. 2019; Mojtabai et al. 2014; Priester et al. 2016; Sinha et al. 2018), while others suggest that both integrated and non-integrated treatments manage similar outcomes and are, thus, viable and effective treatment options (Cleary et al. 2009; Randall et al. 2001).

Thirdly, integrated and non-integrated treatments elicited similar retention rates. However, non-integrated treatment specifically observed significantly better completion rates compared to integrated treatment. These findings were unexpected and not supported by previous research (Morisano et al. 2014; Priester et al. 2016; Sterling et al. 2011). Research claims that the poor engagement, low retention rates, and high drop-out rates evident in non-integrated treatment undermine the potential efficacy of treatment and patient prognosis and outcomes. Therefore, the comparable retention rates observed in both integrated and non-integrated treatments and the significantly higher completion rates in non-integrated treatment may have contributed to the significant yet similar reductions in substance use outcomes.

Consequently, these results indicate that neither treatment model possesses a clear advantage over the other when considering the above-mentioned treatment outcomes. However, both integrated and non-integrated treatments seem helpful and comparably efficacious treatment options. Therefore, it is imperative to look beyond the initial framework of the treatment model and consider the strategies that appear to facilitate change in patients with DD. Ultimately, if effectively treating patients with DD is not about *how* the treatment is delivered, perhaps it is about *what* is being delivered.

The secondary aim of this study was to summarise the strengths and limitations of integrated and non-integrated treatment for patients with DD. Primarily, based on both reported and anecdotal evidence, the CBT modality proved to be the most effective intervention framework for delivering treatment for patients with DD. This finding is consistent with previous studies (Horsfall et al. 2009; Murthy, Mahadevan & Chand 2019; Randall et al. 2001). Furthermore, the results suggest that effective treatment relied on prioritising relational elements such as the therapeutic alliance, fostering feelings of validation and motivation, and maintaining strong lines of communication between treating clinicians. Moreover, the setting where treatment was carried out proved to be a vital facilitator of change. Specifically, in-patient or residential treatment programmes were particularly advantageous for participants with DD due to the reduced influence of environmental risk factors. Several studies acknowledge the same advantage of a controlled setting, citing greater communication and co-ordination between service providers (Cleary et al. 2009; Green et al. 2015; Morisano et al. 2014), reduced psychosocial adversity and exposure to abuse, violence, and illicit substances (Lachman et al. 2012).

In considering the limitations of integrated and non-integrated treatment for patients with DD, the main findings indicated that the advantages of in-patient treatment were lost when operating in an out-patient setting. In particular, the high level of co-ordination and communication fostered among multidisciplinary teams evident in in-patient care was forgone when transitioning to out-patient treatment. Additionally, it is suggested that integrated treatment, although advantageous on a number of fronts, requires multiple service providers with extensive expertise in treating patients with DD. However, this remains a scarce resource. This finding is consistent with the reported difficulties associated with treating patients with DD, as well as implementing integrated treatment (Green et al. 2015; Iudici et al. 2020; McCallum et al. 2015; Morojele et al. 2012; Pasche et al. 2015; Weich & Pienaar 2009). However, only one study within this review identified and spoke to the barriers to change and, as a result, limited the extent to which any generalisations can be made (Coffey et al. 2016).

### Evidence-based recommendations

The final aim of this study was to propose evidence-based recommendations to inform the clinical psychological service delivery of DD-focused treatment in South Africa. Based on the study’s findings, it is recommended that:

- The number of treatment options available for patients with DD be increased, irrespective of the treatment model of delivery being implemented.
- A combination of CBT principles such as mindfulness, self-regulatory skills, cognitive restructuring, and motivational interviewing be implemented.
- Strong lines of communication are maintained, secure therapeutic alliances are developed, and culturally sensitive approaches are cultivated to enhance treatment retention and participant motivation.
- Treatment be conducted in structured settings, such as in-patient or/and community residential programmes, where the influence of environmental risk factors can be limited and the risk of elevated substance cravings and relapse is reduced.

## Limitations of the study and directions for future research

There are several limitations to this study. It must be kept in mind that in conducting a systematic review, there is a potential for fragmented evidence that limits the ability of the results to provide sufficient information on the included studies. The specificity of the eligibility criteria of this review has resulted in the selection of a small yet focused collection of studies. Moreover, the included studies were conducted with samples of predominantly male, Caucasian participants. Therefore, these studies may not be representative of other ethnicities, cultures and socio-economic contexts. The generalisability and applicability of these results beyond the contexts mentioned earlier are, thus, limited. Adapting results from a first world setting to a third world context requires the consideration of several factors.

Several of the studies included in this review specified the exclusion of psychiatric diagnoses such as bipolar mood disorder, schizophrenia, schizophreniform, and schizoaffective disorder in their eligibility criteria. Moving forward, greater efforts to conduct research with these psychiatric populations in mind are necessary to build a comprehensive understanding of DD-centric treatment. Future research should also be directed towards conducting clinical trials that investigate DD-centric treatment’s efficacy in developing countries such as South Africa. These studies are essential to understand the transferability of outcomes achieved in first-world countries and obtain a more comprehensive account of the context-specific barriers patients experience to treatment.

## Conclusion

The findings of this review do not support the enhanced efficacy of integrated treatment over non-integrated treatment or vice versa. However, it was determined that integrated treatment held an advantage over non-integrated treatment in significantly improving psychiatric symptomatology. No significant benefits were found between integrated and non-integrated treatment regarding substance misuse and treatment retention. Further, implementing a combination of CBT-informed principles such as mindfulness, self-regulatory skills, cognitive restructuring, and motivational interviewing proved particularly advantageous. Additionally, establishing and maintaining meaningful therapeutic alliances and strong lines of communication between service providers proved instrumental in participants’ recovery journeys.
